# Using the intraoperative hand held probe without lymphoscintigraphy or using only dye correlates with higher sensory morbidity following sentinel lymph node biopsy in breast cancer: A review of the literature

**DOI:** 10.1186/1477-7819-3-64

**Published:** 2005-09-29

**Authors:** Suk Chul Kim, Dong Wook Kim, Renee M Moadel, Chun K Kim, Samprit Chatterjee, Michail K Shafir, Arlene Travis, Josef Machac, Borys R Krynyckyi

**Affiliations:** 1 Department of Radiology, Division of Nuclear Medicine, The Mount Sinai School of Medicine, The Mount Sinai Hospital, New York, New York, USA; 2 Department of Nuclear Medicine, Albert Einstein College of Medicine of Yeshiva University, and the Montefiore Medical Center, Bronx, New York, USA; 3 Department of Health Policy, The Mount Sinai School of Medicine, The Mount Sinai Hospital, New York, New York, USA; 4 Department of Surgery, The Mount Sinai School of Medicine, The Mount Sinai Hospital, New York, New York, USA

## Abstract

**Background:**

There are no studies that have directly investigated the incremental reduction in sensory morbidity that lymphoscintigraphy images (LS) and triangulated body marking or other skin marking techniques provide during sentinel lymph node biopsy (SLNB) compared to using only the probe without LS and skin marking or using only dye. However, an indirect assessment of this potential for additional sensory morbidity reduction is possible by extracting morbidity data from studies comparing the morbidity of SLNB to that of axillary lymph node dissection.

**Methods:**

A literature search yielded 13 articles that had data on sensory morbidity at specific time points on pain, numbness or paresthesia from SLNB that used radiotracer and probe or used only dye as a primary method of finding the sentinel node (SN). Of these, 10 utilized LS, while 3 did not utilize LS. By matching the data in studies not employing LS to the studies that did, comparisons regarding the percentage of patients experiencing pain, numbness/paresthesia after SLNB could be reasonably attempted at a cutoff of 9 months.

**Results:**

In the 7 studies reporting on pain after 9 months (> 9 months) that used LS (1347 patients), 13.8% of patients reported these symptoms, while in the one study that did not use LS (143 patients), 28.7% of patients reported these symptoms at > 9 months (P < 0.0001). In the 6 studies reporting on numbness and/or paresthesia at > 9 months that used LS (601 patients), 12.5% of patients reported these symptoms, while in the 3 studies that did not use LS (229 patients), 23.1% of patients reported these symptoms at > 9 months (P = 0.0002). Similar trends were also noted for all these symptoms at ≤ 9 months.

**Conclusion:**

Because of variations in techniques and time of assessing morbidity, direct comparisons between studies are difficult. Nevertheless at a minimum, a clear trend is present: having the LS images and skin markings to assist during SLNB appears to yield more favorable morbidity outcomes for the patients compared to performing SLNB with only the probe or performing SLNB with dye alone. These results are extremely pertinent, as the main reason for performing SLNB itself in the first place is to achieve reduced morbidity.

## Background

There is a trend towards minimally invasive surgery in all aspects of breast cancer management. In keeping with this trend is the increasing popularity of sentinel lymph node biopsy (SLNB), which has been shown to result in lower levels of morbidity in the staging of patients with breast cancer as compared to conventional axillary lymph node dissection (ALND) [1-20]. Therefore, it has essentially become the standard of care [21]. Furthermore, SLNB potentially provides even better staging than ALND, as SLNB offers the added benefit of allowing for a more extensive histological analysis of the SNs, by allowing for additional sections through nodes, and by making it possible to use additional immunohistochemical staining methods. These added maneuvers are not routinely performed on nodes obtained from "classical" ALND, since there are too many nodes available for examination from ALND, and testing all of them is not feasible. [22,23].

The role that lymphoscintigraphy (LS) images play in reducing morbidity during SLNB vs. using only the probe without images or using only dye has not been formally examined. Surgeons from ours and other institutions find LS and triangulated body marking (TBM) or other skin marking techniques essential during SLNB [22-26], while others do not find the images or markings helpful [27-29].

Theoretically, an accurate 3-dimentional representation of the number and location of SNs in the body in the form of LS images and TBM reference points or other skin markings, should expedite surgery by allowing a more targeted, minimally invasive approach. This should limit the amount of dissection, and therefore, the level of morbidity. This is an important issue, as the main purpose for performing SLNB is morbidity reduction.

Since no direct studies have investigated the additional reduction in sensory morbidity that LS images and TBM may provide during SLNB, an indirect assessment of this potential was performed by extracting sensory morbidity data on SLNB from studies comparing the morbidity of SLNB to that of ALND [1-20] as described below.

## Methods

The computerized bibliographic databases of PubMed and Medline were searched. The key words used in the search included breast, node, and morbidity. In addition, an extensive manual search and cross-referencing from review and original articles was performed. The global inclusion criteria were prospective and retrospective studies that listed any sensory morbidity related to SLNB in breast cancer patients.

Information on study design, lymphoscintigraphy techniques, time of follow-up, and patients' characteristics were collected from the searched articles. When information was not present in the referenced text, personal communications were initiated with the authors to obtain additional information missing from the text; mainly to establish the use or non-use of LS. Based on the information from the articles and personal communications with the authors, 20 articles were found that met these initial criteria [1-20] (Table 1). Three comparisons were made, based on decreasingly stringent levels of comparative parameters described below.

**Table 1:** Studies with data on sensory morbidity and sentinel lymph node biopsy in breast cancer (ref 1-20).

**Reference**	**Year**	**Journal**	**Country**	**Sample Size**	**Design**	**Lymphoscintigraphy**	**Mean Length of Follow-up**
Schrenk P et al.(1)	2000	Cancer	Austria	35	Prospective	Used	15.4 M
Roumen RM et al.(2)	2001	Br J Surg	Netherlands	100	Prospective	Used	24 M
Burak WE et al.(3)	2002	Am J Surg	USA	48	Prospective	Not Used	15.4 M
Haid A et al.(4)	2002	Breast Ca Res T	Austria	57	Retrospective	Used	18 M
Temple LK et al.(5)	2002	Ann Surg Oncol	USA	171	Prospective	Used	3 M, 6 M, 12 M
Swenson KK et al.(6)	2002	Ann Surg Oncol	USA	169	Prospective	Not Used	1 M, 6 M, 12 M
Haid A et al.(7)	2002	Eur J Surg Ocol	Austria	66	Prospective	Used	Not specified
Leidenius M et al.(8)	2003	Am J Surg	Finland	49	Prospective	Used	2 W
Schijven MP et al.(9)	2003	Eur J Surg Oncol	Netherlands	180	Retrospective	Used	12 M
Blanchard DK et al.(10)	2003	Arch Surg	USA	685	Prospective	Used	2.4 Y
Veronesi U et al.(11)	2003	N Engl J Med	Italy	100	Prospective	Used	6 M, 24 M
Rietman JS et al.(12)	2003	Cancer	Netherlands	66	Prospective	Used*	6 W
Peintinger F et al.(13)	2003	Br J Cancer	Austria	25	Prospective	Used	1 W, 9–12 M
Baron RH et al.(14)	2004	Oncol Nurs Forum	USA	197	Prospective	Used	24 M
Armer J et al.(15)	2004	Lymphololgy	USA	9	Retrospective	Used	9 M
Ronka RH et al.(16)	2004	Acta Oncol	Finland	57	Prospective	Used	12.6 M
Rietman JS et al.(17)	2004	Ann Surg Oncol	Netherlands	66	Prospective	Used*	12 M
Langer S et al.(18)	2004	Am Surg	USA	40	Retrospective	Not Used	51 M
Luini A et al.(19)	2005	Breast Ca Res T	Italy	244	Prospective	Used	6–12 M
Ronka RH et al.(20)	2005	Breast	Finland	43	Prospective	Used	12 M

*Use/method of lymphoscintigraphy unclear, radiotracer used as backup method of finding sentinel node compared to primary dye based technique.

### Initial comparisons

For the initial set of comparisons, parameters of morbidity assessment included; 1) pain in the arm or axilla, and 2) numbness on the operated side and/or paresthesias. A subset of the group of 3 studies not using LS were compared to a subset of the 17 studies that used LS. Studies were matched as closely as possible based on the mean time frames of morbidity assessment, to generate best matched time frames of assessing morbidity.

### Excluded studies and exclusion criteria for the initial comparisons

The study by Schrenk *et al *[1] was initially excluded, because SLNB morbidity data were presented for a mixed group of blue dye only and combined dye/radiotracer procedures.

The study by Temple *et al *[5] was excluded, as a unique morbidity scoring system was employed that could not be adapted in any way for the comparisons. In addition, prevalence data was reported, but the number of patients experiencing symptoms could not be extracted.

One of the two studies by Haid *et al *[7] was excluded, as no data on the timing of symptom assessment were provided. Symptoms tend to decrease over time, and the time of assessment is needed for any meaningful comparisons.

The study of Ronka *et al *[16] presented morbidity data which were strictly confined only to the breast, excluding the axilla where SLNB is performed, and therefore was excluded.

Two studies by Rietman *et al *[12,17], were initially excluded in this constrained comparison because the use of lymphoscintigraphy was unclear [12,17,30-32] and portions of the data in the initial earlier study were in error [12,31,33]. In addition, there are suggestions that a dissection intensive blue dye technique was used as the main method of finding the SN over a more targeted radiotracer/probe method, which appeared to only have a secondary role in finding the SN [12,17,30-32]. These two studies [12,17] were based on the exact same population of patients but at different time points of morbidity assessment. Yet, the later study [17] fails to acknowledge the existence of the earlier study and its results [12].

Luini A *et al *[19] presented morbidity data on a mixed group of ALND and SLNB patients where pure SLNB data could not be extracted. The study was therefore excluded.

We also initially excluded additional studies [2,4,10,14,15,18], because pain and numbness and/or parasthesia morbidity parameters were not assessed at comparable time points to the remaining studies.

For all remaining studies, the criteria for assessing morbidity was chosen as such: either symptoms were present (yes) or absent (no). In studies where a sliding scale of intensity was the method of reporting morbidity symptoms, the percentage of patients fitting into a percent adjusted yes or no group was used [6,20].

After the initial study selection process described above, 7 studies were included in the initial comparative analysis. Among the 7 studies, 5 used LS [8,9,11,13,20] and 2 did not use LS [3,6] (Table 2).

**Table 2:** Initial comparisons. Comparisons of studies at specific time points that met the constrained criteria detailed in the initial comparison (see text).

Morbidity	Mean Length of F/U	Lymphoscintigraphy (+)			Lymphoscintigraphy (-)			p-value
				
		Mor (%)	Total Pt (N)	References	Mor (%)	Total Pt (N)	References	
Pain	≤ 1 m	31.08%	74	8,13	56.52%	161	6	0.0004
	6 m	16.00%	100	11	37.50%	152	6	0.0002
	12–15.4 m	11.71%	222	9,20	28.67%	143	6	< 0.0001
Numbness/Paresthesia	6 m	2.00%	100	11	30.26%	152	6	< 0.0001
	12–15.4 m	4.50%	222	9,20	22.75%	189	3,6	< 0.0001

The 7 studies were grouped into the same morbidity category when data was available. Depending on follow-up periods and sensory morbidity assessment techniques, a total of 5 time periods were established.

The resulting comparable time periods of morbidity assessments for pain were ≤ 1 month, 6 months and 12 to 15.4 months. Likewise, numbness/parasthesia morbidity assessments were at 6 months and 12 to 15.4 months (Table 2).

The statistical analysis was performed using R 2.01 software available in the public domain. Fisher's exact test was used to analyze the nominal variables in the form of frequency tables. A result was considered to be significant only if the P-value was lower than 0.05. All p-values reported are two-sided.

### Secondary comparisons

The 20 studies were then reanalyzed with less stringent time of morbidity assessment criteria. Two time periods were created to include the greatest number of studies in the analysis and resulted in the time periods of ≤ 9 months and > 9 months for pain and numbness/paresthesia (Table 3). This allowed the 6 initially excluded studies from table 2[2,4,10,14,15,18], to be integrated into table 3, resulting in a total of 13 studies included in the secondary comparative analysis. Among the expanded group of 13 studies, 10 used LS [2,4,8-11,13-15,20] and 3 did not use LS [3,6,18] (Table 3). Statistical analysis for Table 3 again utilized Fisher's exact test.

**Table 3:** Secondary comparisons. Comparisons of studies at two time periods that met the criteria detailed in the secondary comparison (see text).

Morbidity	Mean Length of F/U	Lymphoscintigraphy (+)			Lymphoscintigraphy (-)			p-value
				
		Mor (%)	Total Pt (N)	References	Mor (%)	Total Pt (N)	References	
Pain	Acute (≤ 9 m)	22.41%	174	8,11,13	47.28%*	161*	6	< 0.0001
	Chronic (> 9 m)	13.81%	1347	2,4,9,10,11,14,20	28.67%	143	6	< 0.0001
Numbness/Paresthesia	Acute (≤ 9 m)	5.50%	109	11,15	35.58%*	160*	6	< 0.0001
	Chronic (> 9 m)	12.48%	601	4,9,11,13,14,20	23.14%	229	3,6,18	0.0002

* The average morbidity at 1 month and 6 months in article 6. Pain morbidity at 1 month and 6 months are 56.52% (91/161) and 37.50% (57/152), respectively. Numbness/paresthesia morbidity at 1 month and 6 months are 40.63% (65/160) and 30.26% (46/152), respectively.

### Tertiary comparisons

To further maximize the number of studies compared and be as inclusive as possible, additional studies were integrated into a tertiary analysis by relaxation of criteria. These included the study by Schrenk *et al *[1], where the heterogeneity of the data set as noted above was overlooked, and the two articles by Rietman *et al *[12,17], by discounting the issues related to them as noted above and including them in the analysis (Table 4).

**Table 4:** Tertiary comparisons. Comparisons of studies at two time periods that met the revised (relaxed more encompassing) criteria detailed in the tertiary comparison (see text).

Morbidity	Mean Length of F/U	Lymphoscintigraphy (+)			Lymphoscintigraphy (-)			p-value
				
		Mor (%)	Total Pt (N)	References	Mor (%)	Total Pt (N)	References	
Pain	Acute^t ^(≤ 9 m)	22.41%	174	8,11,13	47.28%*	161*	6	< 0.0001
	Chronic (> 9 m)	13.77%	1365	1,2,4,9,10,11,14,20	28.67%	143	6	< 0.0001
Numbness/Paresthesia	Acute (≤ 9 m)	27.43%	175	11,12,15	35.58%*	160*	6	0.1254
	Chronic (> 9 m)	12.56%	677	1,4,9,11,13,14,17,20	23.14%	229	3,6,18	0.0003

* The average morbidity at 1 month and 6 months in article 6. Pain morbidity at 1 month and 6 months are 56.52% (91/161) and 37.50% (57/152), respectively. Numbness/paresthesia morbidity at 1 month and 6 months are 40.63% (65/160) and 30.26% (46/152), respectively.

^t ^This comparison remained unchanged from table 3.

Issues with the remaining articles [5,7,16,19] continued to be too significant to be reasonably included in the tertiary comparison, as no meaningful data could be extracted for the reasons already noted above. Statistical analysis for Table 4 again employed Fisher's exact test.

Among the 20 articles, there were several with similar or partly overlapping patient populations. Specifically the articles of Leidenius M *et al *[8] and Ronka RH *et al *[20], as well as Rietman JS *et al *[12,17] fit this pattern. However, for these articles the times of morbidity assessment were different, so no overlapping of patient populations occurred in any of the comparisons. In one group of articles, Temple *et al *[5] and Baron RH *et al *[14], the former was not included in any of the comparisons for reasons already mentioned above, so no overlapping patient population issues occurred when the latter was included in the comparisons. For the studies of Roumen RM *et al *[2] and Schijven MP *et al *[9], there potentially appeared to have been very minimal overlap of patient populations. The earlier prospective study [2] had data from 90 patients from one hospital, while the later retrospective study [9] had data on 180 patients from the same hospital as well as another hospital, with the later study [9] data collected mainly outside the timeframe of the earlier study [2]. Correspondence with the author of the earlier study [2] suggested no overlap with independent cohorts. Both studies were therefore included in the analysis.

## Results

For studies that met the constrained criteria, for all comparison periods, there was less pain and numbness/paresthesia morbidity reported for studies using LS than those that did not, as shown in Table 2. The differences were highly statistically significant (< 0.001). Similar results were obtained in the secondary comparisons, in which the time of morbidity assessment was segregated into two time periods, allowing more studies to be included in the analysis, as shown in Table 3. For the tertiary comparisons in which inclusion criteria were relaxed as described above to maximize the number of studies included, all comparisons showed lower morbidity when LS was used, but only 3 out of 4 were statistically significant (see Table 4). The p-value for the numbness/paresthesia comparison at ≤ 9 months did not reach statistical significance (0.1254). Globally, for the three levels of comparison (Tables 2, 3, 4), all 12 unique comparisons, using various inclusion/exclusion criteria, showed less morbidity for the LS groups. In 11 out of the 12 unique comparisons, the p-values were highly statistically significant (p-value range < 0.0001 to 0.0004). The results for numbness/paresthesia at > 9 months shown in Table 4 are especially pertinent, as this reflects long term outcomes and includes the largest number of patients, and the greatest number of LS and non LS studies.

## Discussion

The concept that using LS further reduces morbidity over that of using the probe without LS or using dye alone is intriguing to say the least. The analysis we performed suggests that a strong trend exists for additional morbidity reduction in the groups using LS.

The results from this analysis can also be conceptualized from a purely theoretical standpoint; that is: mapping out the location of the SNs in three dimensions prior to incision, knowing the true number of "hot" SNs (or lack of nodes) and the relative intensity and time of appearance to one another should facilitate a targeted surgical approach. Surgeons can plan their approach with this information and adjust their dissection based on the findings of the probe with/without concurrent use of dye.

The results are also supported by the experience of surgeons who have access to high quality LS and skin markings and understand how to use the information. They are of the opinion that both the corresponding images and markings allow for a more directed dissection [23,26].

The studies reviewed in the analysis were not directly trying to evaluate whether LS further reduces morbidity. Had they been, it can be speculated that potentially even better results could have been obtained with LS, especially as the techniques of LS, TBM/skin marking, and SLNB continue to evolve [22-26,34-41], with a renewed focus on accurate anatomical delineation and minimally invasive surgery. In addition, 9 studies that employed LS indicated that they used variable forms of skin marking. Had all studies that employed LS used an updated version of this technique of 3-dimensional SN reference, i.e. TBM, a further morbidity reduction could have potentially been achieved by directing an even more targeted surgical approach [34,36,41].

The greatest limitation of our analysis is that there are only a few studies that did not employ LS (n = 3). Most of the comparisons in the tables were with only a single non LS study [6]. Nevertheless, the results of nearly all comparisons are consistent with results from the specific comparisons that contained 3 non LS studies. The number of studies employing LS was much greater, and provides stable estimates for the proportion having the morbidities of pain and numbness/paresthesias. Because of variations in techniques, time and method of assessing morbidity, direct comparisons between studies are difficult and subject to confounding issues.

The number of nodes removed, use of radiotherapy after SLNB, use of dye in addition to radiotracer, and the harvesting of level III SNs and/or non-axillary nodes could potentially also influence the level of morbidity. These factors were not scrutinized as the information was not consistently available in the referenced articles. For example, information on the biopsy of non-axillary nodes was available only for one reference [8], where 16% (8/49) of SN biopsied were non-axillary. While our study results show an overwhelming advantage for using LS (+) over no LS (-) (essentially a 50% reduction in sensory morbidity at P < 0.0001–0.0003), we are very conservatively describing the results as only a "strong trend" or correlation for the reasons noted above.

Many clinicians dispute any advantages that LS and skin markings may provide in serving the patient during SLNB [27-29,42,43]. The reasons for this could be partly explained by the great variability in the quality of LS studies that are performed [23,27,28,34-37], and/or perhaps by difficulties on the part of the surgeons in integrating the information provided by LS and skin markings into their surgical techniques during SLNB. Surgical techniques need to be addressed in the pursuit of morbidity reduction. Training tools for surgeons have been developed that have the potential for integrating the information from LS and skin markings, with the goal of performing minimally invasive surgery [38,40].

When the surgeons have a 3-dimensional concept and image of the location of SNs prior to surgery, they are then able to fine tune their surgical approach. An example of this ability to fine tune surgical approach can be applied to a hypothetical case of an upper outer quadrant breast lesion. Initially, the surgeon may plan to attempt to reach the SN via the tumor excision site in an attempt to reduce morbidity. However, when provided with LS images and skin markings the surgeon might realize that this is not the optimal approach from a standpoint of morbidity reduction, and would be able to plan an alternative approach that further minimizes morbidity. It is conceivable that more experienced, better informed surgeons are more likely to utilize LS because of its benefits, and that these surgeons perform a more delicate dissection resulting in less morbidity. However, the exact contribution of LS vs. the skill level of the surgeons that directly contributes to this reduction in morbidity can not be independently deduced from the data in the articles.

Based on this analysis, the surgical evaluation of the sentinel node in the axilla should utilize LS, TBM or other skin marking techniques, and a probe guided dissection in order to reduce sensory morbidity.

## Conclusion

Though no randomized studies exist that directly ask this question, our literature analysis suggests that lymphoscintigraphy imaging is a very useful tool in further reducing morbidity during sentinel lymph node biopsy.

## Competing interests

The author(s) declare that they have no competing interests.

## Authors' contributions

**SCK **contributed to data analysis, design, and preparation of manuscript.

**DWK **contributed to data analysis, design, and preparation of manuscript.

**RMM **contributed to data analysis, design, and preparation of manuscript.

**CKK **contributed to data analysis, design, and preparation of manuscript.

**SC **contributed to data analysis, design, and preparation of manuscript.

**MKS **contributed to data analysis, design, and preparation of manuscript.

**AT **contributed to data analysis, design, and preparation of manuscript.

**JM **contributed to data analysis, design, and preparation of manuscript.

**BRK **developed the initial concept and contributed to data analysis, design, and preparation of manuscript.

All authors read and approved the final manuscript.
